# Physician’s role(s): Conceptual redesign and implementation of the lecture series “Introduction to clinical medicine” for the promotion of professional identity formation based on the physician’s roles of the CanMEDS framework

**DOI:** 10.3205/zma001826

**Published:** 2026-03-23

**Authors:** Moritz Schumm, Sandra Apondo, Antonius Schneider, Pascal O. Berberat

**Affiliations:** 1 TUM Medical Education Center, Department Clinical Medicine, TUM School of Medicine and Health, Technical University of Munich, Munich, Germany; 2 Institute of General Practice and Health Services Research, Department Clinical Medicine, TUM School of Medicine and Health, Technical University of Munich, Munich, Germany

**Keywords:** CanMEDS, professional identity formation, professionalism, lecture, medical education

## Abstract

**Aim:**

The CanMEDS framework of the Royal college of physicians and surgeons of Canada and its role profile were originally contrived for the training of specialist physicians. But they also deliver an ideal teaching content to engage undergraduate medical students with the examination of their own professional development as well as with the institutional and social expectations of physicians. Integrating the physician’s roles of CanMEDS into teaching can thus be recognized as an element of professional identity formation. As an example, the article presents a lecture series of the TUM Medical Education Center that implements the CanMEDS framework in its current version from 2015 into its teaching.

**Method:**

The article showcases the lecture series “Introduction to clinical medicine” that underwent a reconceptualization and gradual redesign starting with the 2022 winter semester. The general structure as well as content-specific outlines of the lecture series are presented in line with the respective roles of the CanMEDS framework. Special attention is payed to the applied teaching methods of the lecture series (impulse-based teaching, panel discussions, photo competition).

**Results:**

After currently two iterations assessments deliver a so far very positive résumé. Comprised of direct gradings as well as text comments by the students the evaluation offers first insights into the reception of the contents and methods (interactivity, reflective practice and duration).

**Conclusion:**

The positive feedback regarding the redesign so far is an encouragement for further adjustments in the chosen direction. A next step is to establish the used methods on a broader basis in lectures that involve elements of professional identity formation.

## Zusammenfassung

**Zielsetzung:**

Ursprünglich für die ärztliche Weiterbildung entworfen, bietet das CanMEDS-Modell des Royal College of Physicians and Surgeons of Canada und seine Rollenprofile einen idealen Lehrinhalt, um auch Studierende zur Auseinandersetzung mit ihrem ärztlichen Werden sowie mit den institutionellen und gesellschaftlichen Erwartungen an sie anzuregen. Die Rollen des CanMEDS in die Lehre einzubeziehen kann entsprechend als ein Element der professionellen Identitätsentwicklung und -förderung (Professional Identity Formation) fungieren. Der Artikel stellt exemplarisch ein Vorlesungsprojekt des TUM Medical Education Center vor, anhand dessen das CanMEDS-Modell in der aktuell gültigen Version von 2015 in die Lehre integriert wurde.

**Methodik:**

Vorgestellt wird die für das Wintersemester 2022/23 neu ausgerichtete und seitdem sukzessive umgestaltete Vorlesungsreihe „Einführung in die klinische Medizin“. Präsentiert werden die allgemeine Struktur und inhaltliche Ausrichtung der Reihe entlang der Rollen des CanMEDS. Insbesondere wird auf die angewandten Lehrmethoden (impulsbasierte Lehre, Podiumsdiskussion, Fotografiewettbewerb) der Lehrveranstaltung eingegangen.

**Ergebnisse:**

Nach aktuell zweimaliger Durchführung lassen die Evaluationen, die sich aus den direkten Bewertungen durch die Studierenden sowie Freitextkommentaren zusammenfügen, ein sehr positives Résumé ziehen, das auch erste Schlaglichter auf die Inhalte und Methoden erlaubt (Interaktivität, Reflexionsangebot und zeitlicher Rahmen).

**Schlussfolgerungen:**

Die positiven Rückmeldungen über die bisherigen Umstellungen ermutigen zu weiteren Anpassungen. Geplant ist v. a., die angewandten Methoden auf breiterer Basis in Vorlesungen zu etablieren, die Elemente zur professionellen Identitätsentwicklung beinhalten.

## 1. Introduction

In its current version, the mission of the CanMEDS framework of the Royal college of physicians and surgeons of Canada – initially contrived for the training of specialist physicians – is: “[T]o articulate a comprehensive definition of the abilities needed for all domains of medical practice and thus provide a strong foundation for medical education” [1]. The framework’s continuous revisions since its first iteration in the 1990s further articulate the conviction to optimally prepare aspiring physicians – in the face of a rapidly changing world – for the challenges and opportunities of contemporary clinical realities [1], [2]. To this end, the framework follows its tried and trusted method: Relying on a competency-based understanding of medical practice, a catalogue of “key competencies” and “enabling competencies” is developed, which combines these competencies to units and presents them as distinct roles of which the medical profession is comprised. The “CanMEDS cloverleaf” [2] can be regarded as the most prominent expression of this process.

The framework was initially conceptualized with specialist physicians in mind. Nevertheless, it can also gain relevance for undergraduate medical students when it comes to professional identity formation as part of medical education [3], [4], [5]. It is not only delivering insights into the required professional capabilities that physicians have to master, but also into the expectations of society and the different roles that give shape to medicine’s professional practice. As such the CanMEDS framework also forms one of the foundational pillars of the “Ärztliche Kompetenzrollen” (“medical competency roles”) of the German National competence-based catalogue of learning objectives for medicine (see: [https://nklm.de/zend/objective/list/orderBy/@objectivePosition/modul/200553/obsolete/no], last accessed May 28, 2024). Understood as stimuli for reflection, the role profiles of CanMEDS have the potential to offer undergraduate medical students an important support for the development of their individual identity as a physician: They can bring together competence-based, social as well as individual aspects of the medical profession and subject them to critical examination so that students can not only anticipate but also review and discuss them. They can contrast their own expectations of the medical profession with the ones put forward by the medical institution and the ones held by society to develop a clearer understanding of their own role as a physician, the challenges they face as well as the opportunities for individual adaptation available to them.

In contrast to these potentials the CanMEDS framework is to a very large extent and up to this day almost completely unknown to students. Therefore, a concept for a lecture series was developed at the TUM Medical Education Center that puts the different roles of CanMEDS center stage as guidance for structure as well as main teaching content. The article presents how the lecture series was utilized to directly implement the CanMEDS framework into medical teaching. It shows the general structure, the main contents and their orientation, and the methods by which students were introduced to the CanMEDS roles. Insights from evaluations so far and an outlook on further plans for development complete the article.

## 2. A lecture series on the physician’s roles of the CanMEDS framework

### 2.1. General structure

To represent the CanMEDS framework in medical teaching the lecture series “Introduction to clinical medicine” was redesigned for the winter semester 2022/23. The series consists of eleven individual teaching units and functions as part of the so-called “interdisciplinary lecture 1” (IDL-1) which is given for third year students. The whole IDL-1 comprises 52 teaching units and is focused on the teaching of pathophysiological basics. “introduction to clinical medicine” initially followed the intention to offer students at the beginning of the clinical stage an overview of the cross-sectional and interdisciplinary topics of clinical practice. The series’ title is similar to the one of a mandatory pre-clinical internship for medical students called “introduction to clinical medicine (with patient presentation)”, but does not refer to it in any direct way. The already given diversity of topics addressed by the original lecture series offered the foundation for the representation of the CanMEDS roles and competencies next to the regular teaching of clinical expertise. The CanMEDS framework in its 2015 version [1] was used for the implementation into the lecture series. Additionally, expected changes and enhancements of the framework by the next revision planned for 2025 were also considered. “Planetary health” [6] and a strengthened emphasis on “physician humanism” [7] as potential new main foci of the framework promise to connect well with the lecture series’ approach. 

To keep the established interplay of the approximately 20 teachers involved in the lecture series intact, a step by step and as smooth as possible implementation of the redesign presented a further essential task. The whole project has therefore to be seen as a still ongoing work in progress. After completion of the second installment during the winter semester 2023/24 a presentation of the general conceptualization and its realization is already productive for review. Table 1 (Tab. 1) gives an overview over the redesigned structure of the lecture series, its adaptation of CanMEDS’ different roles as well as the underlying competencies within the framework that are also functioning as learning objectives for teaching. 

Some aspects of this conceptualization need special consideration: First, double representation of roles by different lectures can be accredited in general to a more exhaustive attention paid to the associated competencies. The role “scholar” in the very first session represents an extension of the originally addressed aspects of CanMEDS. Here, the focus is set on students themselves as recipients of the CanMEDS framework. The very last lecture of the series is dedicated to the “medical expert” as the centrally staged intersection of all roles of CanMEDS. All other nine lectures were attuned to the CanMEDS framework in different ways: Some already existing topics could be taken over into the new leitmotif with only smaller changes. Especially the topics “medical communication” and “physicians as health advocates” were already part of the curriculum and could directly be identified with the roles of “communicator” and “health advocate”. Other topics were also left unchanged to a great extent, but needed to be related to a specific CanMEDS role. The topics “medical scientificity” was reoriented towards the role of “scholar”, the topic “physicians as practical ethicists” towards central aspects of the role of the “professional”.

By these changes all petals of the CanMEDS framework were integrated into the lecture series and inform its structure as well as content. Still, further changes are in planning. Leaving the general conceptualization untouched, these changes will focus on a further successive methodological realignment. This follows the conviction that lectures with focus on stance and personal attitude cannot – if they want to have an impact – rely solely on chalk-and-talk teaching. Instead, teaching methods have to be applied which involve the students directly into the teaching [8]. Consequently, students relate to teaching goals not just as abstract facts, but as experienced phenomena that have to be thought through and critically reviewed. Equally relevant was an expected increase of the lectures’ appeal for students when innovative formats also deliver an invitation for active participation. One basic didactical element in this regard was the general encouragement of all teachers to use a personalized access to all topics and also to address the students’ individual stance towards the subject in interactive phases of the lecture. By this, and besides all the teaching goals that rely on objective facts, the lecture aims to also highlight the personal and subjective view of students on central aspects of clinical practice. Further, to allow for continuity and a consistent atmosphere, the lecture series is moderated throughout by the chair of medical education as well as the chair of general medicine. Finally, new teaching methods were introduced into the series of which three are presented in the next section.

### 2.2. Teaching methods for conveying physician’s roles

#### 2.2.1. Teaching based on impulses (“narrative medicine”)

Although there are methodical deviations, the use of impulses in the lecture series as a didactical tool is based on the teaching approaches brought forward by “narrative medicine” [9], [10]. The central idea is to start a conversation about a specific topic by first presenting and analyzing an impulse that is non-medical but instead an artefact of cultural history. Results of such analysis can enable a new perspective on a subject that can subsequently be transferred and applied to the medical realm. This produces a reflexive move that can be understood as “a kind of skills lab in which one can experiment, speculate, try out and train.” ([11], translation by MS).

The very first lecture of the series offers an example. The focus is set on the role of “scholar” with special attention payed to students’ own position within and perception of medicine. As already mentioned further above, this focus transcends the frame set by the CanMEDS framework. Here emphasis is put not only on an understanding of “scholar” as the academically trained expert, but also as the person that is becoming one. Thus, the students’ perspective becomes an important part of the teaching. In this context, it is important to keep in mind that student’s attendance of the lecture series is situated at the beginning of the clinical stage. Using this background students are shown a clip of the short movie “hopptornet” (Axel Danielson, Maximilien van Aertryck, S 2016), a film staged in a documentary mode to depict people and their behavior when standing on the top of a ten meter tower: Some show their worries when looking down into the pool far beneath, others are gathering their courage. Some are focused, others try to distract themselves or seem to be torn between opposing feelings. Some abort and move down the ladder, others risk the jump.

**Figure 1:**
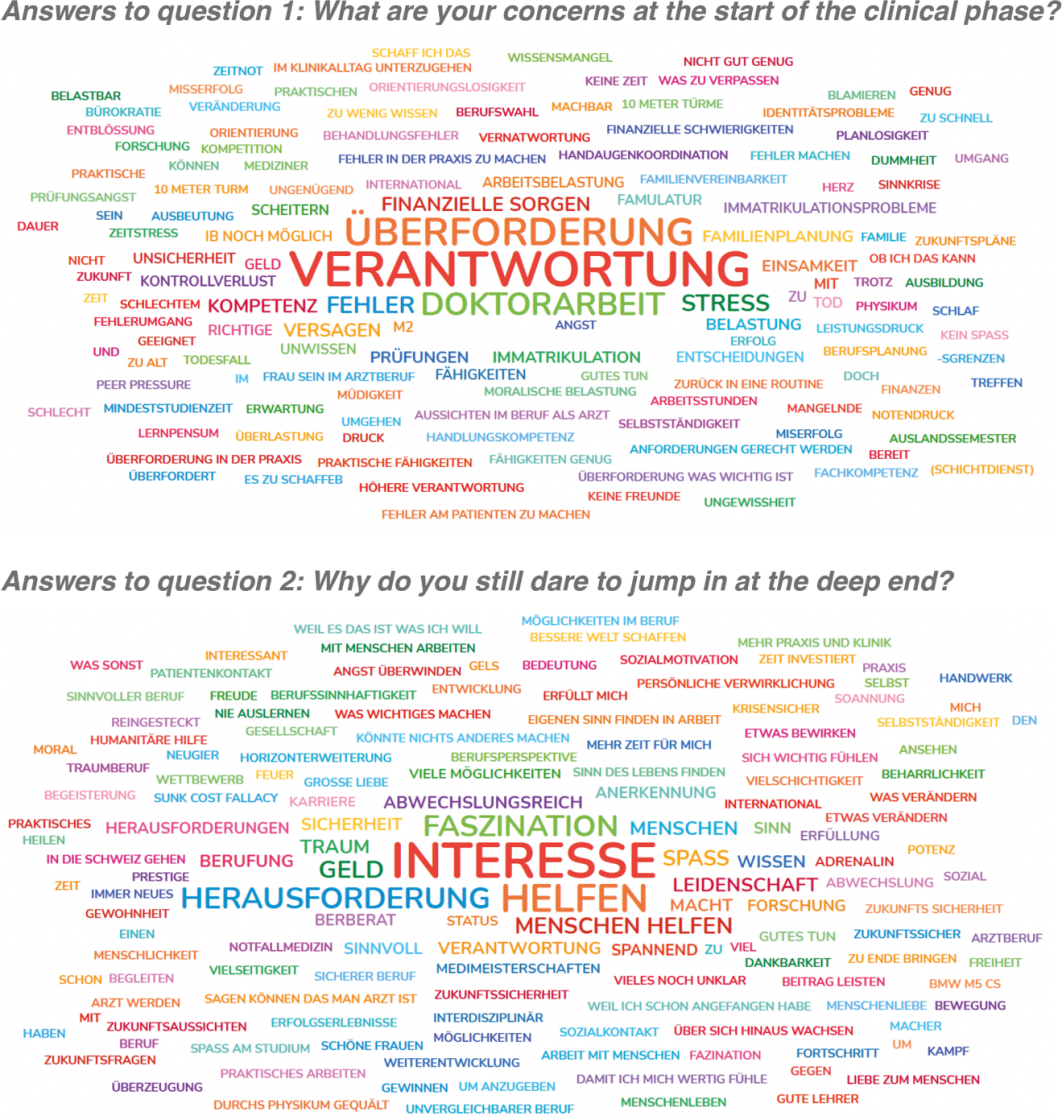
Original (German) answers to the questions from the first, impulse-based teaching unit of the lecture series “Introduction to clinical medicine” (winter semester 2023/24)

The clip gives reason for laughs during the screening which seems to be due to the affective structure of the depicted situation. This interpretation is based on the answers students give to the first two questions they are asked: “Who has ever stood themselves on the top of a ten meter tower? How did it feel?” Results are collected by giving the question directly to the auditorium. Answers to the questions represented an audience well-acquainted with the situation so that the very own experiences enriched the viewing of the film clip with a specific emotional background and meaning. But the target of the impulse was not only reminiscence. The main target was to connect it with the present and the beginning of a new phase in student’s study program. This connection was introduced by two further questions for the audience. Instead of a continuation of the direct dialog, students were now asked to submit their answers by using an online polling tool. The results were fed in the beamer presentation in real time to make them directly visible for everyone. The questions were: “What are your concerns at the start of the clinical phase?” and: “Why do you still dare to jump in at the deep end?” Following this procedure the lecture collects a wide spectrum of positive as well as negative associations as its results (see figure 1 (Fig. 1)). Introduced by a playful impulse the lecture offers students the chance to reflect their own current position as well as their personal expectations of their professional future. 

#### 2.2.2. Panel discussion and talk with students

The second important format introduced by the redesign of the lecture series are panel discussions that allow to present topics not as abstract concepts but in direct connection with medical practice. To this end, representatives of different clinical sectors are invited to get into dialogue with each other on a specific subject. The discussion is organized and structured by questions of the moderator. Further, students are offered the possibility to participate by allowing questions from the audience. The last third of the lecture is reserved for this Q&A. The method as well as its incorporation is shown here by describing one exemplary lecture.

Two lectures are held as panel discussions so far. One of them is concerned with the topic “physicians as part of a team”. Four representatives from TUM University Hospital constitute the interprofessional panel: two physicians of different medical disciplines, the head of nursing science of TUM University Hospital as well as a deputy head nurse. After introducing the guests to the audience moderation asks each panel member questions concerning the work within a team, its significance for clinical work as well as examples for worst and best practice. The course of the dialogue and the coverage of topics are dependent on the dynamic of the discussion, but normally revolve around some non-varying main areas. These are: the efficiency of interprofessional interaction, a reciprocal understanding of the professions and the inclusive integration of every professional expertise involved. All of these aspects are also reflected in the CanMEDS framework under the competencies 1.1–1.3 as well as 2.1 und 2.2 of the role of “collaborator” [1].

For the last third of the lecture students are invited to participate in the discussion with their own perspective on the topic. The inclusive setup of the format in combination with the integration of students by asking for their input is regarded as a special incentive to attend the lecture. Participating in the panel discussion is only possible by physical attendance and looses this benefit when watched as a video recording (joining lectures online was not offered for the lecture series).

#### 2.2.3. Photo competition

A photo competition was established as a special format that spans the whole lecture series. The task is presented to students during the first lecture: they are asked to – literally – picture the role physicians fulfill and to submit the result as a photography. Time is given till nearly the end of the lecture series. The task can be handled by students individually or in a group of up to five participants. The competition is also functioning as a required test performance to successfully pass the lecture series. As the whole lecture series is about deeply subjective aspects addressing personal attitude as well as stance the format of a photo competition as test performance was chosen to give (lasting) expression to individual perception and creative adaptation. At the same time the term competition was taken seriously: A jury of staff members of the lecture series and TUM Medical Education Center held a vote to pick three winners and a wider selection of particularly successful works.

A presentation of the jury’s selection is giving form to the last lecture of the series with the title “Becoming a physician – being a physician: A conclusion”. Photographs are sorted beforehand by the lecture’s teachers for presentation slides according to their depiction of different roles of the CanMEDS framework. During the lecture the photographs are shown and discussed in the context of the respective CanMEDS roles. The lecture’s focus is not only relying on the accordance of the selected works with a specific role of the CanMEDS framework. Equal attention is payed to the actual creativity in its execution. Some pictures are using visual puns. Others take a critical look at contemporary medicine and their own role within it. Emphasis is put, for example, on self-care in combination with the roles of “professional”, “leader”, or “communicator”. Some photographs are dedicated to specific aspects and details, whereas others view medicine in general terms. Dying and death or the omnipresence of existential experiences for medical professionals are represented in many pictures and related to different roles of the CanMEDS framework – be it “professional”, “communicator”, “manager”, “leader”, or “health advocate”. A further frequently depicted topic is interprofessional work to reflect the roles of “manager”, “leader”, as well as “scholar”. An overview of the submitted works can be found on the Instagram channel of the LET ME program (@lettered_medicine).

The photographs that were selected by the jury are presented here when authors’ consent was provided. The photographs shown in the final lecture are working themselves as impulses. A panorama is assembled of the physician’s roles created by students themselves to illustrate the horizon of the “medical expert” as CanMEDS’ central role. Finally, there is of course also an announcement of the first three picks by the jury – each accompanied by a price – to give the last lecture a festive conclusion.

## 3. Assessment by students and outlook

The lecture series was redesigned with the intention to acquaint students with the physician’s roles and their significance for clinical everyday life as well as for their own professional self-image. To this end, CanMEDS was chosen as an already completely worked out framework that is also an essential source for the German National competence-based catalogue of learning objectives for medicine which is still in development. Further, the framework’s specific visualization as flower petals allows for a particular feel and intuitive comprehensibility very well suited for teaching 

**Figure 2:**
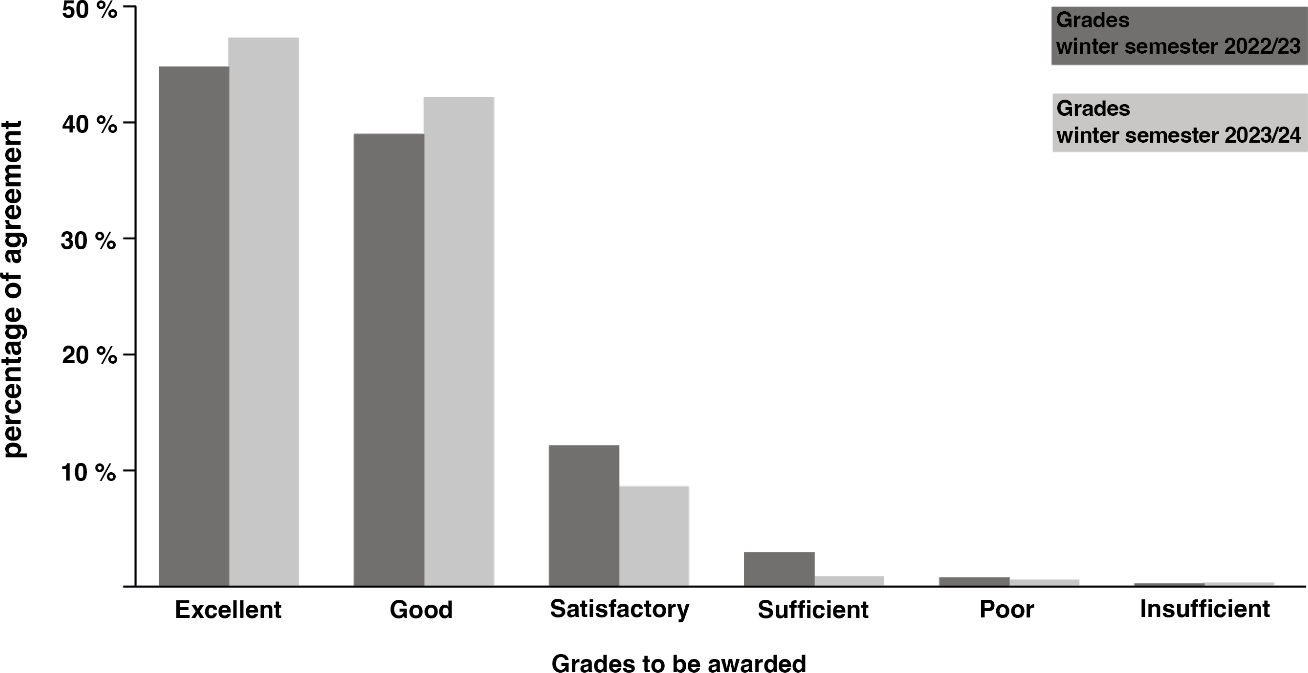
Grades for the lecture series “Introduction to clinical medicine” for the winter semesters 2022/23 and 2023/24 by participating students

Redesign of the lecture series was launched for the winter semester of 2022/23 and has seen – up until now – two installments. A standard assessment was used to evaluate the lecture series. It asks students to give a grade to the individual lectures while an additional comment section allows for written out feedback. A more comprehensive assessment specifically evaluating the redesigned lecture series is pending. But so far gathered results still allow to draw first conclusions (see figure 2 (Fig. 2)).

Some aspects featured in the comment section further concretize the reasons for the assessment results. The very first lecture, for example, was praised for its application of teaching methods emphasizing interactivity (“good interactivity”, “I love the lecture’s different approach with its interactive parts”), while at the same time the unusual format and its open-ended structure were criticized: “Not really much hard facts for organization of medical specialties and what to expect of them, but instead a lot of commonplaces that are not really useful.” On a more general level the space given to reflection by the lectures was received positively: “A good mix of self-reflection and inspiration”, “an excellent chance to reflect on “becoming a physician”, “very good to not only have theoretical, medical content, but also lectures that span over diverse topics and encourage contemplation and reflection.” (All translations in this paragraph by MS).

One critical aspect concerning especially the panel discussions that was also voiced in the assessment of the winter semester 2023/24 referred to the limited time spent on such a format. A longer duration was demanded. Attendance figures were also surveyed and resulted in numbers in line with the average attendance of the complete IDL-1. They don’t allow for more nuanced insights into the potential of innovative formats as an incentive to attend lectures.

Assessment of the so far made changes up till now draws a positive picture of the redesigned lecture series and its focus on the CanMEDS framework. Even if innovative formats so far could not underpin an intended increase of appeal, positive postings in the comment section form the strong majority. Following this outcome additional alterations of the lecture series are planned to further the implementation of the formats and methods represented here to allow for more direct participation with students in lectures.

Technical University Munich only teaches the clinical stage of the medical curriculum. Having the lecture series right at the beginning of the clinical stage was therefore an important concern. But such a course could also be integrated into the first pre-clinical semester to enable the reflection of the roles of the CanMEDS framework at the start of studies with the outlook of a heightened productivity for students.

## Authors’ ORCIDs

- Moritz Schumm: [0009-0008-2663-7815]
- Sandra Apondo: [0009-0000-4546-435X]
- Antonius Schneider: [0000-0002-2847-8626]
- Pascal O. Berberat: [0000-0001-5022-5265]

## Competing interests

The authors declare that they have no competing interests. 

## Figures and Tables

**Table 1:**
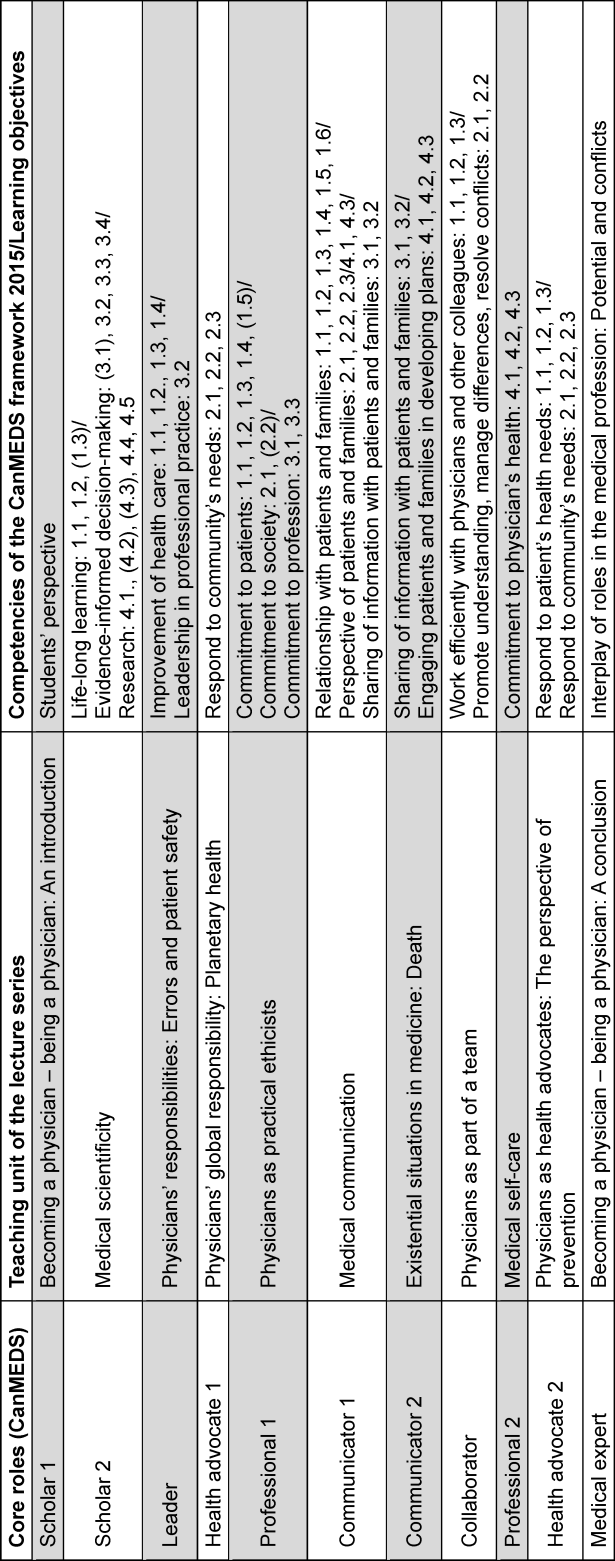
Thematic schedule for the lecture series “Introduction to clinical medicine” based on the role specifications of the CanMEDS framework 2015 [1]
